# Botulinum neurotoxin type A in the interdisciplinary treatment of sialorrhea in adults and children—update and practice recommendations

**DOI:** 10.3389/fneur.2023.1275807

**Published:** 2023-12-13

**Authors:** Wolfgang H. Jost, Tobias Bäumer, Andrea Bevot, Ulrich Birkmann, Carsten Buhmann, Maria Grosheva, Orlando Guntinas-Lichius, Rainer Laskawi, Sebastian Paus, Christina Pflug, A. Sebastian Schroeder, Björn Spittau, Armin Steffen, Bernd Wilken, Martin Winterholler, Steffen Berweck

**Affiliations:** ^1^Parkinson-Klinik Ortenau, Wolfach, Germany; ^2^Institute of Systemic Motor Science, CBBM, University of Lübeck, Lübeck, Germany; ^3^Department of Neuropediatrics and Developmental Medicine, University Children’s Hospital Tübingen, Tübingen, Germany; ^4^Department of Neurology, Schluckambulanz, GFO Clinics Troisdorf, Troisdorf, Germany; ^5^Department of Neurology, University Hospital Hamburg-Eppendorf, Hamburg, Germany; ^6^Department of Otorhinolaryngology, Head and Neck Surgery, Medical Faculty, University of Cologne, Cologne, Germany; ^7^Department of Otorhinolaryngology, Jena University Hospital, Jena, Germany; ^8^Department of Otorhinolaryngology, University Hospital Göttingen, Göttingen, Germany; ^9^Department of Neurology, GFO Clinics Troisdorf, Troisdorf, Germany; ^10^Department of Voice, Speech and Hearing Disorders, University Hospital Hamburg-Eppendorf, Hamburg, Germany; ^11^Clinic for Child Neurology and Social Pediatrics, Child Center Maulbronn, Maulbronn, Germany; ^12^Anatomy and Cell Biology, Medical School OWL, Bielefeld University, Bielefeld, Germany; ^13^Department for Otorhinolaryngology, University of Lübeck, Lübeck, Germany; ^14^Department of Pediatric Neurology, Klinikum Kassel, Kassel, Germany; ^15^Department of Neurology, Sana Hospital Rummelsberg, Nuremberg/Schwarzenbruck, Germany; ^16^Specialist Center for Pediatric Neurology, Neurorehabilitation and Epileptology, Schön Clinic, Vogtareuth, Germany

**Keywords:** sialorrhea, botulinum toxin, botulinum neurotoxin type A, BoNT/A, IncobotulinumtoxinA, hypersalivation, quality of life, practice recommendations

## Abstract

Sialorrhea is defined as a chronic excessive flow of saliva from the mouth, often with adverse consequences for health and quality of life of patients. In addition to currently used non-drug treatment and systemic drugs, intraglandular Botulinum Neurotoxin A (BoNT/A) injections have been examined in case studies, controlled trials and clinical practice. Two pivotal Phase III trials recently led to market approval in the USA and EU for IncobotulinumtoxinA [Xeomin®, IncoBoNT/A, *Clostridium botulinum* neurotoxin type A (150 kD), free from complexing proteins, Merz Pharmaceuticals GmbH] for treatment of chronic sialorrhea in adults and pediatric patients. This review provides a multidisciplinary approach to discuss the current state of sialorrhea therapy as well as benefits and current limitations of BoNT/A injections. A consensus regarding treatment recommendations made available to physicians in Germany in 2022 has now been updated here for presentation to an international audience. This review provides a framework including a flow chart for patient selection, recommendations for dosing and the injection process, as well as a discussion of therapeutic goals, long-term benefits and safety aspects. This review is aimed at supporting physicians in developing multidisciplinary and individualized treatment approaches to achieve optimal benefits for patients.

## 1 Introduction

Sialorrhea is defined as a chronic excessive flow of saliva from the mouth that can have a serious impact on a patient’s health and quality of life. It is one of the symptoms that can accompany several neurological disorders. Current therapies include systemic drugs, even though often used off-label, as well as non-drug treatments. In addition, intraglandular Botulinum Neurotoxin A (BoNT/A) injections for patients with sialorrhea have been explored for many years, in clinical practice as well as in case studies and controlled trials (1–13). Two pivotal Phase III trials showed good efficacy and a favorable safety profile for IncobotulinumtoxinA [Xeomin®, IncoBoNT/A, *Clostridium botulinum* neurotoxin type A (150 kD), free from complexing proteins, Merz Pharmaceuticals GmbH] (1–3). This evidence recently led to its market approval in the USA and EU for treatment of chronic sialorrhea in adults and pediatric patients.

Following these studies and subsequent approvals, the experts involved in this review discussed the current state of sialorrhea therapy in clinical practice, the benefits of BoNT/A injections, and the current limitations and obstacles of this treatment. The multidisciplinary approach of these discussions was essential to assess the issues from different perspectives and to define best practices. Consensus regarding the treatment recommendations was reached by evaluating the clinical trial data, by discussing the current treatment guideline “Hypersalivation” from the German Society of Oto-Rhino-Laryngology, Head and Neck Surgery (DGHNO-KHC) (14), and based on the broad practical experience of the contributing authors. These recommendations were made available to physicians in Germany in 2022 (15, 16) and have now been updated here for presentation to an international audience.

BoNT/A therapy for patients with sialorrhea is highly individualized and should be a collaborative effort between experts from different medical and therapeutic disciplines. The aim of this review is to provide a framework for developing multidisciplinary treatment concepts for sialorrhea on a case-by-case basis and to support treating physicians in their treatment decisions for optimal patient outcomes.

## 2 Etiology and characteristics of sialorrhea

In most cases chronic sialorrhea is caused by excessive collection of saliva in the mouth due to impairments in swallowing or in orofacial functions. A less common reason for hypersalivation is an overproduction of saliva in the glands due to a side effect of certain medications which is not addressed in this text here. The term “hypersalivation” is often used synonym for sialorrhea. Increased salivation is normal in the first 4 years of life and is regarded as pathological after that (17–19).

Sialorrhea can be a symptom of various neurological disorders, including neurodegenerative Parkinson’s syndromes, dementia, apraxia, Huntington’s disease, cerebrovascular diseases (e.g., stroke), inflammatory diseases of the central nervous system (e.g., multiple sclerosis, encephalitis, meningitis), motor neuron diseases (e.g., amyotrophic lateral sclerosis), brain tumors, craniocerebral trauma, intoxication, and cerebral palsy. Sialorrhea is also seen in disorders of the peripheral nervous system like caudal cranial nerve disorders and Guillain-Barré syndrome. Sialorrhea can also have other causes, e.g., head and neck cancers, defects after surgery or after radiation of head and neck or of the upper gastrointestinal tract. Furthermore, patients in palliative care and ventilated and/or tracheotomy patients can experience sialorrhea. Drug-induced sialorrhea can occur with parasympathomimetics (e.g., pyridostigmine) and neuroleptics (especially clozapine, risperidone, haloperidol, aripiprazole).

The consequences of chronic troublesome sialorrhea are two-fold. Firstly, sialorrhea can cause health issues, e.g., due to saliva causing skin maceration on face and hands or saliva entering the respiratory tract, which can lead to serious events like aspiration pneumonia. These issues are particularly troublesome because they add to the symptoms that patients experience due to their underlying neurological disorders. Secondly, sialorrhea has a severe impact on the quality of life of patients and caregivers, leading to social stigma, embarrassment, and withdrawal from social life. In the authors’ experience, patients and caregivers are sometimes neither aware of the extent of the sialorrhea symptoms and consequences nor of the treatment options that can provide relief. Physicians should actively discuss the issues with patients and/or their caregivers to determine whether treatment is required.

**Figure 1:**
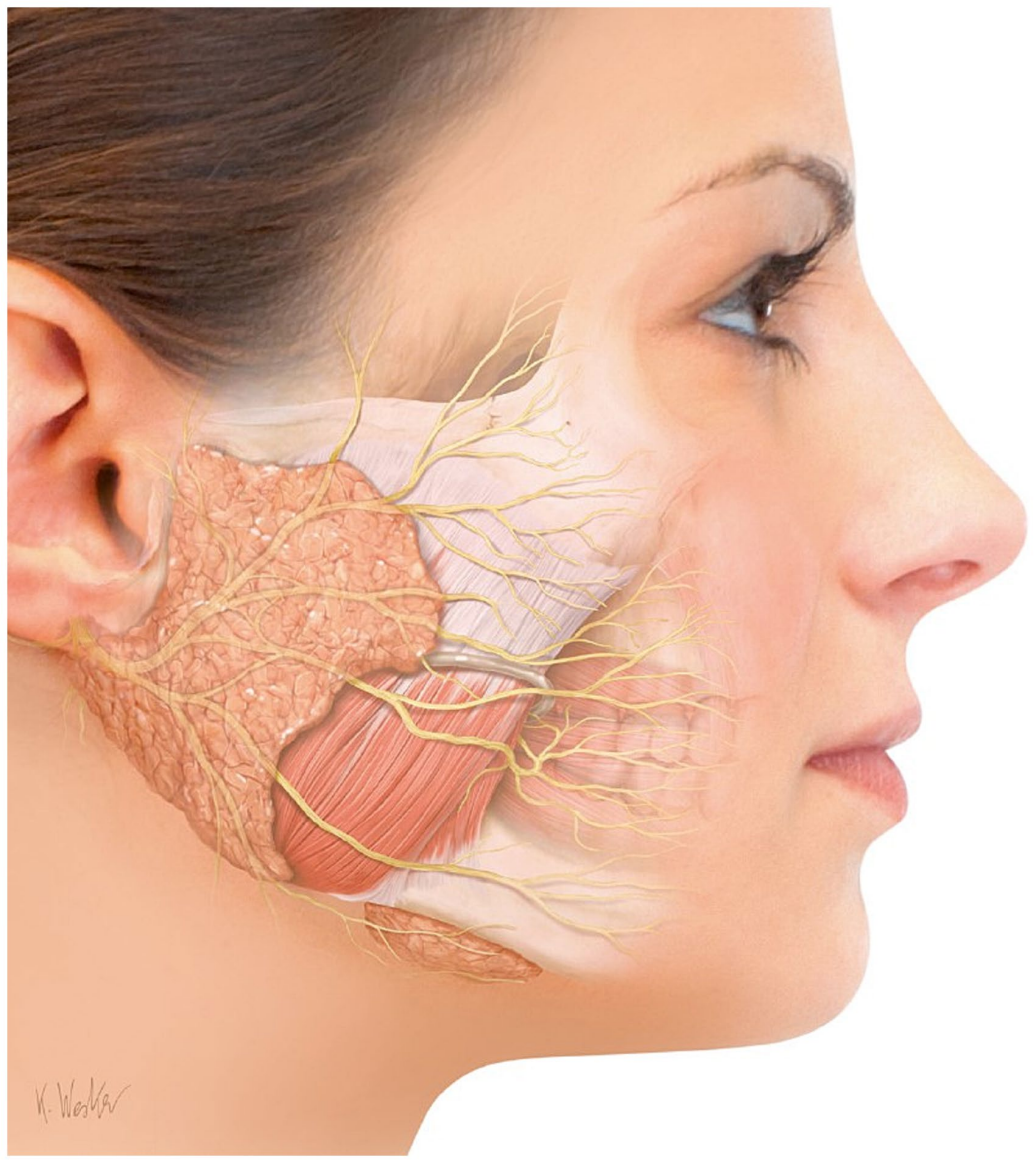
Schematic illustration of the location of the salivary glands. Actual position and size of the salivary glands show patient individual variability. Therefore, ultrasound-guided injection is recommended. (Modified according to Jost W., Atlas der Botulinumtoxin-Injektion © KVM—Der Medizinverlag, Berlin).

Several drugs and non-drug treatments for sialorrhea are available, albeit with known side effects particularly for systemic acting drugs. Now that IncoBoNT/A gained approval by the regulatory authorities both for adult and pediatric patients, it is a viable alternative or addition to existing therapy schemes. Unlike other drugs, BoNT/A acts locally, directly in the salivary glands. The most important salivary glands are the three major salivary glands, i.e., the paired parotid, submandibular, and sublingual glands (Figure 1). They are located in the neck, around the oral cavity. The parotid glands are the largest glands and extend vertically from the zygomatic arch to the anterior border of the sternocleidomastoid muscle and horizontally from the mastoid process to the parotid duct via the masseter muscle. They secrete saliva into the oral cavity via the parotid duct which opens through the buccal mucosa opposite of the second upper molar tooth. The sublingual glands are located in the floor of the mouth below the tongue. The submandibular glands are positioned inside the submandibular space below the mylohyoid line partly covered by the mandible. The sublingual and submandibular gland have a common duct, entering the oral cavity at the floor of the mouth to both sides of the frenulum of the tongue (20–22). The submandibular glands produce by far the largest volume of saliva (~70%), followed by the parotid gland (~25%). Salivary secretion is controlled by the autonomic nervous system and is an important first step of food digestion. It contributes to the degradation of complex carbohydrates, the wetting of solid food, and the release of nutrients and flavors. Furthermore, saliva ensures a continuous rinsing of oral surfaces, controlling biofilm development and, thus, protecting teeth from caries. The average production of saliva in adults is 1–1.5 L per day. The production of salvia can be stimulated and increased up to five times from the basal production value by eating or smelling food (23–25). While the submandibular glands are mainly responsible for the basal mucous and serous salvia, the parotid glands are responsible for the serous stimulated component (26, 27).

## 3 Botulinum Neurotoxin use for treatment of sialorrhea

Botulinum toxins are high molecular weight protein complexes derived from *Clostridium botulinum* bacteria. The molecules’ neurotoxin section is the pharmacologically active component. Botulinum Neurotoxins act by binding selectively to receptors on peripheral cholinergic nerve endings, where the toxin is internalized and ultimately inhibits the release of the neurotransmitter acetylcholine at the neuroglandular junction in a dose-dependent manner (28, 29). This leads to a reversible decrease in saliva production and—in many cases—to a clinically relevant improvement in sialorrhea.

Botulinum Neurotoxin Types A [IncobotulinumtoxinA (Xeomin®), OnabotulinumtoxinA (Botox®), AbobotulinumtoxinA (Dysport®)] and B [RimabotulinomtoxinB (Neurobloc®)] are used for medical purposes. For sialorrhea treatment, the toxin is injected into the large salivary glands, which leads to a decrease of salivary secretion. The recent clinical trials and subsequent marketing approvals for IncoBoNT/A in sialorrhea treatment confirm the effectiveness that physicians have observed for off-label use in clinical practice for almost two decades (30–32).

## 4 Clinical trials

Substantial evidence has been obtained in clinical trials to show efficacy and safety of BoNT (types A and/or B) for treatment of sialorrhea. Trials enrolled patients with Parkinson’s syndrome, amyotrophic lateral sclerosis, early childhood brain damage, ear/nose/throat tumors, tracheal cannulas and ventilation, and dysphagia of various types. In most trials, efficacy was evaluated by quantitative measurements of saliva production over time, accompanied by subjective assessments of patients’ quality of life (8–10, 12, 13, 19, 33–35). While efficacy and safety is comparable both with BoNT/A and BoNT/B, there is evidence of a clinically relevant tendency to form antibodies for serotype B (5, 33, 35).

Two Phase III trials using IncoBoNT/A were pivotal for gaining marketing approval for this compound (Xeomin®) from the Food and Drug Administration (FDA) and European Medicines Agency (EMA) for sialorrhea in adults (in 2018/2019) and in pediatric patients (in 2020/2021).

The first of these trials (SIAXI) was a randomized, placebo-controlled, double-blind study, treating adult patients with either 75 units or 100 units of IncoBoNT/A (74 patients per group), while 36 patients received placebo. The most frequent underlying conditions of the study patients were idiopathic Parkinson’s syndrome, atypical Parkinson’s syndrome, stroke, and traumatic brain injury. The BoNT/A injections in this trial were distributed among the parotid glands (60% of injection volume) and submandibular glands (40% of injection volume), leading to the approved treatment recommendations. Significant improvements in sialorrhea were seen during the 16-week follow-up phase after injection with 100 units BoNT/A, based on the unstimulated salivary flow rate (uSFR) and the subjective Global Impression of Change scale (GICS). Side effects (adverse events assessed as related to treatment) were observed in around 9% of patients in each treatment group (including placebo), none of which were serious (1). The placebo-controlled first cycle of the SIAXI study was followed by up to three further open-label, dose-blinded injection sessions (75 units or 100 units IncoBoNT/A) up to 48 weeks. The results of this extension period showed sustained efficacy with continued good tolerability and high patient adherence. For around 4% of patients treated with the 100 units dose, dysphagia was reported as a side effect (2). A clear advantage of using ultrasound guidance for injections (optional in this trial) was not observed. Marketing approval for sialorrhea treatment in adults was subsequently granted for the higher of the two dosages (100 units), as this showed a longer and somewhat stronger efficacy with a good safety profile.

The SIAXI trial was followed by a similarly designed Phase III trial for pediatric patients aged 2–17 years (SIPEXI). A total of 220 patients (body weight ≥ 12 kg) were included and received pre-specified body weight-adapted doses in the range of 20–75 U IncoBoNT/A (around 2 U/kg) or placebo. Patients weighing ≥ 30 kg received a fixed dose of 75 U. After consultation with the regulatory authorities, none of the patients under 6 years of age received placebo for ethical reasons. Ultrasound guidance of the injection procedure was mandatory in this trial. The body weight-dependent dosing scheme and the distribution among the parotid and submandibular glands, which was based on the SIAXI trial and clinical experience, proved adequate in this trial and was the basis for the approved dosing recommendations for children and adolescents (see Table 1). The SIPEXI trial showed good results, comparable to the SIAXI trial in adults. In the 16-week follow-up period after the first injection session, IncoBoNT/A was superior to placebo, both regarding the reduction in salivary flow rate and the subjective improvement measured on the GICS. The subsequent open-label extension period with up to three further injection sessions over up to 48 weeks confirmed efficacy and safety. An additive effect was observed over time, with treatment effects increasing with repeated injection cycles. This was more pronounced than in the SIAXI trial. Treatment was well-tolerated throughout the trial. In the first injection cycle, 2 patients aged 6–17 years experienced side effects after IncoBoNT/A treatment, none of which were serious. During the open-label extension period, 8 patients (5.5%) experienced side effects, including four patients with dysphagia. Tolerance in the 2–5-year-olds was similarly good; only one patient experienced side effects which were injection site reactions (3).

**Table 1:** Dosing scheme for weight-dependent dose determination for pediatric patients.

Body weight	Parotid gland, each side		Submandibular gland, each side		Total dose (both glands, both sides)
	*Total dose (units) per gland*	*Volume per injection*	*Total dose (units) per gland*	*Volume per injection*	
≥12 and <15 kg	6 U	0.24 mL	4 U	0.16 mL	20 U
≥15 and <19 kg	9 U	0.36 mL	6 U	0.24 mL	30 U
≥19 and <23 kg	12 U	0.48 mL	8 U	0.32 mL	40 U
≥23 and <27 kg	15 U	0.60 mL	10 U	0.40 mL	50 U
≥27 and <30 kg	18 U	0.72 mL	12 U	0.48 mL	60 U
≥30 kg	22.5 U	0.90 mL	15 U	0.60 mL	75 U

### 4.1 Patient selection for BoNT/A treatment

Sialorrhea treatment is highly individual due to the condition’s varied etiology and extent. No one-fits-all scheme can be prescribed, and physicians must take several factors into consideration when selecting patients for BoNT/A treatment. Generally, adult patients and pediatric patients aged 2 years or older (≥12 kg body weight) with troublesome sialorrhea can be considered for treatment. A patient’s subjective experience of the severity of clinical symptoms and their impact on quality of life must be at the core of any therapeutic decision. Standardized assessment tools and questionnaires can be used to evaluate quality of life and the level of social interactions (see section on therapeutic goals below). The treatment with IncoBoNT/A is approved for sialorrhea of neurological etiology. However, it is of importance to include the extent of the sialorrhea and its direct consequences into the treatment decision. It must be noted that when assessing pediatric patients with sialorrhea, their intellectual skills must be judged carefully, as these are often underestimated due to outward appearances.

The next step is a physical examination which should be extensive and multidisciplinary. It should determine or clarify an underlying neurological condition, assess muscle function, anatomy, and dental issues. Specialists should be consulted as needed, including otorhinolaryngologists, speech therapists, and phoniatricians.

Patients’ eligibility for BoNT/A treatment can be limited for various reasons and the treating physician must assess the circumstances carefully before making treatment decisions. Cases of suspected (micro-)aspiration, respiratory problems when eating, excessively long meals, or repeated pneumonia must be examined in detail. The German guideline “Hypersalivation” from the DGHNO-KHC (14) recommends a screening of swallowing ability and a phoniatric or ear/nose/throat examination, including endoscopic diagnostics [e.g., fiber-optic endoscopic evaluation of swallowing (FEES)] and/or X-ray videofluoroscopy (36–42). In this context, recent developments show the value of real-time magnetic resonance sequences as a promising option allowing an assessment that does not involve active participation by the patient (43, 44). As dysphagia is the most frequent cause of sialorrhea, a targeted assessment of any swallowing issues is essential before proceeding with treatment decisions. The authors recommend active questioning of the patient and/or caregiver according to the checklist provided in Table 2. Treatment of patients with pronounced dysphagia must be considered carefully, as dysphagia is also a rare side effect of BoNT/A injections (1–3). An unintended exacerbation of the existing dysphagia or issues due to the change in salivary flow may be problematic in such patients. This depends on whether the dysphagia is caused by disturbed secretion leading to aspiration of superfluous saliva or by difficulties in swallowing food or medication without residues in mouth and throat, whereby a reduction of salivary flow can be detrimental. The reduction of salivary flow by BoNT/A can increase the risk of dental issues due to increased biofilm formation. Patients with existing disorders like caries or gingivitis should receive dental treatments before and/or during BoNT/A therapy and adequate prophylaxis. Dental check-ups for children during the treatment with BoNT/A are recommended in general. In cases of drug-induced sialorrhea, a reduction, discontinuation or replacement of the causative drug should be discussed before considering the patient for BoNT/A treatment. Patients on anticoagulant therapy with vitamin K antagonists, new oral anticoagulants or high-dose heparin treatment should, if possible, pause their anticoagulant therapy before receiving BoNT/A injections. If this is not possible, the patient must be monitored for approx. 30 min after the injections to rule out complications due to hematoma formation in the floor of the mouth. Before treatment with IncoBoNT/A the contraindications must be observed as per product labeling; patients with known hypersensitivity to any of the product components or with an infection at the proposed injection site cannot be treated. The effect of IncoBoNT/A injections can be increased when combined with irradiation treatment of head and neck area (including salivary glands) and/or with simultaneous use of anticholinergics, which should be avoided. Furthermore, due to lack of data, IncoBoNT/A should not be used during pregnancy and lactation.

**Table 2:** Dysphagia anamnesis—checklist for active questioning.

**Direct symptoms:**	**Indirect symptoms:**
Swallowing salvia or while drinking and eating Frequent coughing/clearing of throat during or after eating/drinking Gurgling voice/so-called “wet voice”	Recurring pneumonia (pneumonia, recurring bronchitis, unclear fever) Unwanted weight loss Food “does not go down” Food residues in mouth/throat Chest pain while eating

**Figure 2:**
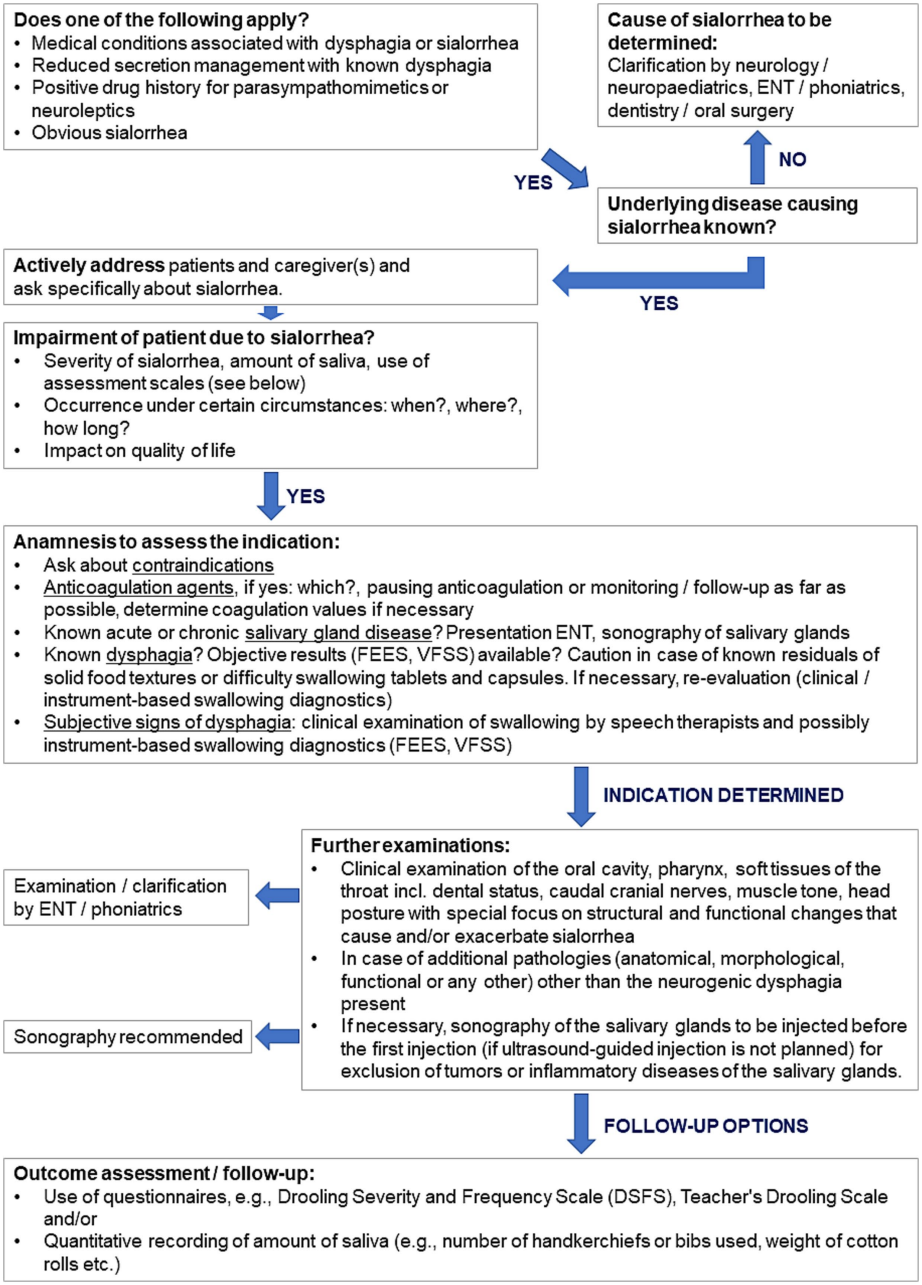
Flow chart for selection of patients for BoNT/A treatment. DSFS, Drooling Severity and Frequency Scale; ENT, Ear nose throat physician; FEES, flexible endoscopic evaluation of swallowing; VFSS, videofluoroscopic swallowing study.

The authors have developed a flow chart to guide physicians in selecting patients who can benefit from BoNT/A treatment (Figure 2). The chart is intended to close any gaps in diagnostics and to support well considered treatment decisions. Depending on the outcomes of the assessments, patients are identified as either eligible for BoNT/A treatment, eligible with limitations, or non-eligible.

### 4.2 Dosing recommendations

The approved total dose of IncoBoNT/A for the treatment of sialorrhea in adults is 100 units (reconstituted in 2 mL sterile saline 0.9%). This dose is distributed among the salivary glands in a pre-defined manner: the left and right parotid glands are injected with 30 units each (0.6 mL solution), and the left and right submandibular glands are injected with 20 units each (0.4 mL solution). The recommended time point for re-treatment is after 16 weeks, based on clinical trial experience (1).

For pediatric patients aged 2–17 years, dosing is weight-dependent and must be (re)calculated for each injection session. The dosing recommendations presented in Table 1 are based on the dosing scheme from the SIPEXI trial, where body weight-adapted dosing proved appropriate for reaching efficacy and safety goals. The recommended dosing scheme results in a total dose of around 2 U/kg body weight (3).

Both from a medical and a legal perspective, IncoBoNT/A (Xeomin®, Merz Pharmaceuticals GmbH, Germany) is recommended for treatment of sialorrhea, as it is currently the only approved BoNT/A product marketed for this indication.

### 4.3 Administration of BoNT/A injections and ultrasound guidance

As described above, an assessment of a patient’s suitability for BoNT/A injections, as well as the correct determination of the required dose are essential before treatment. As a next step, patients (and/or their caregivers) need to be informed and advised in detail about the potential benefits and risks of the treatment and about the actual procedure. Pain medication or sedation can be offered as necessary. The preparation of the BoNT/A solution must be performed according to the package leaflet.

Intraglandular injections of BoNT/A are performed transcutaneously into the paired parotid and submandibular glands, resulting in four injection sites among which the prescribed total dose is distributed. The transcutaneous route of administration has proved the safest option (45). As the parotid glands are larger than the submandibular glands (46, 47), they should be injected with a larger volume of IncoBoNT/A, as described above (14). The sublingual glands are not treated with BoNT/A due to their anatomic location and their minimal contribution to the overall amount of saliva produced.

While intraglandular injections can be performed using anatomical landmarks, the use of ultrasound guidance for a safe injection procedure is an important aspect of the latest treatment recommendations (48). It can minimize the risk of adverse events like swallowing difficulties due to local diffusion of the toxin into the floor of the mouth or pharyngeal musculature (6). This is of utmost importance in patients with sialorrhea, as they are often already dealing with the burden of their neurological disease. Sonographic guidance is mandatory when treating pediatric patients due to their smaller size and their variability in location. It is also highly recommended for treatment of adult patients. In the SIAXI trial, about half of the patients were injected with ultrasound guidance, which was optional in this trial. No difference in efficacy was seen between patients who received ultrasound guidance and those who did not (1). This indicates that orientation by anatomical landmarks may be sufficient to obtain a treatment effect. However, the results do not allow reliable conclusions regarding the difference in injection accuracy with and without sonographic needle guidance. This was explored in a small study with two injectors and six body donors, which showed a significantly higher injection accuracy for injections into the submandibular glands under ultrasound guidance; the difference was smaller for injections into the parotid glands (49). The submandibular glands are particularly difficult to inject due to their variation in size and due to their occasionally unexpected location in some patients (47). Thus, the German guideline “Hypersalivation” from the DGHNO-KHC (14) recommends sonographic guidance for effective and safe intraglandular injections of BoNT/A.

**Figure 3:**
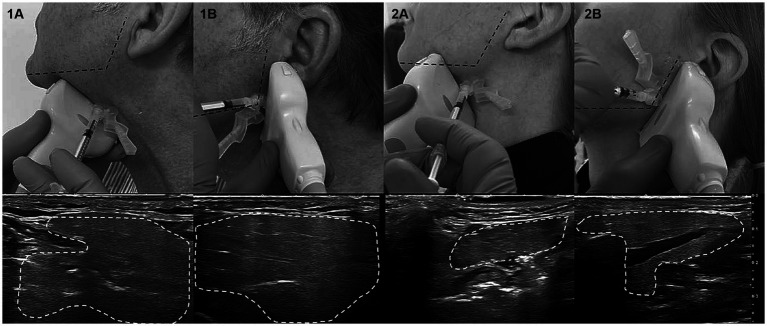
Demonstration of ultrasound-guided injection in subject 1 and 2 on the left side, respectively. **(A)** Submandibular gland, **(B)** Parotid gland. (Copyright Tobias Bäumer, Institute of Systemic Motor Science, CBBM, University of Lübeck).

From a practical perspective, ultrasound needle guidance does not require high-end sonographic equipment. The authors recommend a linear ultrasonic transducer (from 7 MHz) as used for vascular sonography. The anatomic landmarks for the salivary glands are a useful starting point for localizing the glands by ultrasound and assessing their position and extent (48, 50). The landmark for finding the parotid glands is midway between the tragus and the angle of the jaw. Starting at the landmark about 2 cm below the mandible, midway between the mandibular angle and the tip of the chin, the submandibular gland is found dorsally toward the mandibular angle (51). The glands clearly contrast with the surrounding tissue due to their homogenous structure and clear demarcation. Once the gland is located, the ultrasound transducer should be placed centrally on the gland. The injection can be performed “in plane” (guiding the needle along the longitudinal axis of the transducer) or “off plane” (along the transverse axis). When injecting only one point, the needle is usually placed in the center of the gland and not near a duct; when injecting multiple times (usually no more than 2–3), the injection points are spaced appropriately across the gland. The transducer can be aligned horizontally or vertically during the injection and can be held at a slight angle to visualize the needle tip in the planned injection depth. The variability regarding the exact position of the parotid glands is low, allowing for similarly accurate injections with and without sonographic guidance. However, the position and size of the submandibular glands show notably more variability, making precise injections according to anatomical landmarks more difficult and potentially leading to injections at the edge or outside the gland. A schematic of the ultrasound procedure is shown in Figure 3.

### 4.4 Therapeutic goals and long-term benefits

The therapeutic goals for patients treated with BoNT/A for sialorrhea are highly individual, reflecting the heterogeneous patient population. The treating physician should discuss realistic goals with a patient and/or caregiver and agree on how to evaluate treatment outcomes. The overall goal is always the substantial reduction of sialorrhea, ideally decreasing salivary flow to a normal level. This has a direct impact on the extent of troublesome physical health issues, but also—and often most importantly—on quality of life. Patients and caregivers may experience social difficulties and stigmatization, embarrassment in public, and subsequent social withdrawal even within families. A reduction of (visible) hypersalivation can make a crucial difference regarding these aspects and can make situations like functional therapy sessions more comfortable. Furthermore, eating difficulties can be ameliorated, tracheostomy care can be facilitated, and aspiration pneumonia and skin maceration can be prevented. Thus, in essence, the improvements after BoNT/A treatment must be sufficiently large to lead to a notable improvement of patients’ everyday life.

A treatment effect can be expected within a week after IncoBoNT/A injections, the maximum effect is seen around 1 month after injection and the duration of effect is around 3 to 4 months (1). In cases where a short-term reduction of salivation is required, the time to effect may be bridged with careful use of anticholinergic medication (usually off-label).

Especially after a patient’s very first BoNT/A therapy session, close monitoring of treatment effects and possible side effects is advisable, e.g., by using questionnaires, patient diaries, or validated scales. For patients who experience notable benefits and improvements in their physical and psychosocial burden at the latest after the first two or three treatment sessions, a long-term re-treatment schedule should be developed. The results from the pivotal Phase III trials in adults and pediatric patients, which both included up to four treatment cycles, showed that IncoBoNT/A treatment effects increased with repeated injections (2, 3). In light of this evidence, physicians should neither immediately discontinue treatment nor increase the BoNT/A dose if the first cycle’s results disappointed. The authors recommend to re-inject with the planned dose and measure the effects over two or three cycles before evaluating the overall outcome. It can be challenging to convince patients and/or caregivers that this is useful, in particular if they experienced the injection procedure as stressful. In the long run, control visits should be scheduled around 4 months after injections to assess the need for re-treatment. From the authors’ clinical experience, it appears that re-injection intervals can be prolonged over time for many patients because treatment effects are sustained for longer, whereby the reason for this is not entirely clear yet.

Reliable outcome assessment tools are the foundation for all the above considerations. Improvements in quality of life are measurable by using standardized patient-reported outcome tools to follow-up with patients and/or their caregivers. In the authors’ experience, patient diaries are highly useful in theory but require high levels of compliance by the patient. A more direct assessment, also of the frequency of sialorrhea, can be performed by the treating physician using scales like the Teacher’s Drooling Scale (52), the Drooling Severity and Frequency Scale (DSFS) (53, 54), or a Global Impression of Change (GICS) assessment. These instruments can be administered at follow-up visits to examine the treatment effect or possible progression of sialorrhea. Direct measurements of amounts of saliva (e.g., by collecting and weighing fluid or soaked bibs/cotton rolls) are not used regularly in clinical practice due to their methodical weakness and difficulty of reproduction.

### 4.5 Safety aspects and troubleshooting

IncoBoNT/A treatment for sialorrhea is safe and well-tolerated with few side effects, as seen both in clinical trials and in clinical practice (1–3). The risk of side effects may be minimized by injecting with ultrasound guidance only, by using aspiration before injecting to avoid accidental application into a blood vessel, and by adhering to the recommended precautions and contraindications, as outlined above.

As laid out in the summary of product characteristics for IncoBoNT/A, possible side effects that can occur in connection with the injection procedure are local pain, inflammation, paresthesia, hematoma, and bleeding at the injection site. Pain or anxiety caused by the injection can lead to vasovagal reactions such as transient hypotension, nausea and syncope. Side effects that may occur in connection with the effects of IncoBoNT/A in adults with sialorrhea include dry mouth, dysphagia, paresthesia, speech disturbance, thickened saliva and subjective taste disturbance. Dysphagia occasionally occurs in children and adolescents. Thickened saliva, dry mouth, mouth pain and caries can also occur and should be taken into account. Some other typical issues that have been seen in clinical practice are outlined in this section, accompanied by the authors’ recommendations.

Patients may be disappointed by insufficient treatment effects. In such cases, it is advisable to perform several injection cycles at regular intervals and evaluate the outcomes after repeated treatments. If necessary, the BoNT/A dose may be gradually increased. The objective and subjective judgment of treatment effects can differ, and it is important to discuss realistic treatment goals with patients/caregivers before therapy starts. Treatment effects can be limited due to misplaced BoNT/A injections and sonographic needle guidance is also recommended for this reason. The patient’s underlying neurological condition may have deteriorated or progressed, leading to a worsening of symptoms that was not compensated for by BoNT/A. A secondary non-response to treatment due to the development of neutralizing antibodies to IncoBoNT/A was not observed in the clinical trials but cannot be ruled out as a rare (and potentially product-specific) cause of lack of treatment effect.

Patients may experience a dry mouth or xerostomia with highly viscous mucus. This can occur particularly in patients with tracheostomy tubes. Such cases require additional care to optimize salivary secretion management and provide relief. Fluid intake should be increased, oral care should be intensified (e.g., using creams, dexpanthenol sprays, glycerine sticks), and inhalation can be helpful, even in combination with a cough assist device or a vibrating vest in rare cases.

Patients may experience the occurrence or worsening of a swallowing disorder. In such cases, a potential progression of the underlying disease or newly emerging symptoms or diseases must be considered. Endoscopic swallowing diagnostics (FEES) are useful to determine such causes and to rule out the injection or diffusion of BoNT/A into the muscles of the floor of the mouth or pharynx as a causative factor. Changes in a dyskinetic movement disorder are also possible factors for changes in swallowing issues, particularly during child development phases, e.g., puberty. A decrease in BoNT/A dose should be considered if insufficient salivary flow is observed, leading to problems with food intake, due to potential over-treatment.

Patients may experience sialadenitis or mucositis of either infectious or non-infectious origin. Here, oral care must be intensified, analgesia can be offered, and antibiotic treatment or a consultation with an otorhinolaryngologist may be required.

Finally, patients may develop hematoma after injection. This must be monitored and, if possible, any ongoing anticoagulation therapy should be paused. After an event of post-injection hematoma, another occurrence should be prevented by compression of the injection site in the next treatment cycle.

The aim of the current dosing recommendations is a good balance between benefits and risks of BoNT/A treatment. If side effects are observed, physicians should lower the total dose and observe whether efficacy can still be achieved, while reducing adverse events. Based on clinical experience of the experts it may be considerable to start the treatment of adults by administering a total dose of 75 units instead of the recommended 100 units. Likewise, the total dose for pediatric patients can be lowered if clinically appropriate.

### 4.6 Comparison with other treatments

BoNT/A treatment for sialorrhea is a comparatively new therapeutic option. Other drugs have been used for years, mainly atropine, scopolamine, and glycopyrrolate, three antagonistic anticholinergics (17, 55–66). Of these, only glycopyrrolate is approved for sialorrhea treatment in pediatric patients with severe symptoms. The main issues with these systemic treatments concern their safety. They bear the risk of serious anticholinergic side effects, although glycopyrrolate has shown an acceptable safety profile without severe neurological complications. In randomized controlled trials, patients experienced a benefit from short-term use of glycopyrrolate and the safety risks were deemed acceptable (67, 68).

Invasive non-drug treatments like surgery are available, with their obvious disadvantages and risks. Surgical salivary gland interventions primarily include submandibulectomy and several forms of duct interruptions and ligatures, as well as relocation of the ducts of the large salivary glands. The latter requires careful consideration regarding a potentially increased risk of aspiration (53, 69–78). External radiation has been validated as another effective treatment option for sialorrhea (79–84), but its side effects and carcinogenic potential must be considered. Radiotherapy can reduce hypersalivation in cases where treatment with BoNT/A was not successful (85), and conversely, BoNT/A may be used for treatment of post-radiogenic hypersalivation (86). Other non-drug treatments like individualized functional therapy (e.g., swallowing training for patients with dysphagia), orthodontic treatments, or physiotherapy can also provide relief (87–91).

The main advantages of BoNT/A treatment over the abovementioned therapeutic options are first and foremost in the safety profile, in conjunction with good efficacy. BoNT/A is administered locally and acts directly in the salivary glands. This is an important advantage for patients for whom the systemic side effects of anticholinergics bear a high risk, including elderly patients with cognitive impairments, patient with hallucinations, children with intellectual disability, and patients with cardiovascular risk factors. In contrast to several techniques in swallowing programs, no therapy compliances or cognitive ability to perform the maneuvers are needed.

## 5 Conclusion

Chronic sialorrhea as a symptom of many neurological diseases can be a severe burden for patients and should receive adequate medical attention. It affects not only patients’ physical health but can severely impact quality of life by leading to social stigmatization, embarrassment, and withdrawal from social life. Previously available therapy options have shown only limited therapeutic success, for example due to patients’ lack of cooperation in swallowing therapy, persisting symptoms despite optimized drug therapy for Parkinson’s syndromes, or unacceptable side effects after the use of systemically acting anticholinergics, which are often used off-label.

A BoNT/A product has received medical approval for sialorrhea treatment of adults and children, giving physicians the possibility for application in a wide range of diseases. The approvals were based on results from two recent Phase III trials that showed that IncoBoNT/A is an effective and well-tolerated treatment for sialorrhea, also for repeated use. This confirms the benefits observed in previous smaller studies and in years of using BoNT/A off-label in clinical practice. It is recommended to perform the injections according to the approved dosing schemes, i.e., with 100 units of IncoBoNT/A, dissolved in 2 mL saline of 0.9% concentration and distributed among the paired parotid and submandibular glands, ideally using ultrasound guidance. When treating children, doses are determined according to body weight and ultrasound guidance is essential. BoNT/A treatment can lead to a clinically relevant decrease in sialorrhea and to meaningful improvements in quality of life. The long-lasting treatment effect supports good, long-term treatment adherence.

BoNT/A therapy for sialorrhea is highly individual and should be a multidisciplinary effort. In many cases, BoNT/A is the adequate first line treatment. The patient selection process for BoNT/A therapy is largely a diagnostic task to assess a patient’s risk factors and to determine the level of monitoring required during and after BoNT/A therapy. The group of patients who cannot be treated is comparably small. Treatment should be combined with other therapies like functional therapy, orthodontic treatments, or physiotherapy, depending on the individual patient’s needs. Especially important is the combination of BoNT/A treatment with intensive dysphagia therapy, with the latter supporting the diminishing effects of the injections in the intervals between BoNT/A treatment sessions. The combination of BoNT/A application and functional dysphagia therapy is rated highly useful by the experts editing this review and detailed assessments of such schemes in controlled trials would be valuable.

In summary, the authors of this review have outlined practical details of planning and executing BoNT/A treatment and provided recommendations based on their broad and longstanding experience. The aim of this review is to encourage and support physicians to use BoNT/A therapy and to achieve optimal therapeutic outcomes.

## Author contributions

WJ: Writing – review & editing. TB: Writing – review & editing. AB: Writing – review & editing. UB: Writing – review & editing. CB: Writing – review & editing. MG: Writing – review & editing. OG-L: Writing – review & editing. RL: Writing – review & editing. SP: Writing – review & editing. CP: Writing – review & editing. ASc: Writing – review & editing. BS: Writing – review & editing. ASt: Writing – review & editing. BW: Writing – review & editing. MW: Writing – review & editing. SB: Writing – review & editing.
